# Medical News

**Published:** 1856-07

**Authors:** 


### THE MEDICAL EXAMINER.

PHILADELPHIA, JULY, 1856.

### MEDICAL NEWS.

### THE LOUISVILLE REVIEW.

The above is the title of a new journal edited by Prof. S. D. Gross and T. G. Richardson, M. D., published every alternate month in Louisville, Kentucky. In offering a new Journal to the profession, the editors use the following language: "We cannot conscientiously say there is a call for another, notwithstanding the hiatus that has been produced by the suspension of the Western Journal of Medicine and Surgery, the oldest and the ablest publication of the kind that the valley of the Mississippi has ever produced. But, as stated in a circular issued some weeks since, we have undertaken the enterprise 'as a labor of love,—love for the labor and for the glorious profession in which we are actively engaged ;' and this is our only apology to that class of Pre-Adamites who are startled from their ancient propriety, and consider themselves personally affronted by every new thing, and this, our only introductory to the liberal minded progressive few who heartily welcome every honest endeavor which has for its object the diffusion of knowledge and the progress and improvement of a noble science." They inform their readers also that it is their intention to devote their best energies particularly to the Review Department, by which means they expect to keep them *au courant* with the host of new medical works "that are shouldering" each other into the notice of the profession, not neglecting, however, those sections usually devoted to original communications and selections.

We most sincerely and heartily wish success to this new enterprise, and judging from the specimen number we have received, we think it not only fully deserves but will obtain it. It occupies 144 pages, is handsomely printed, and contains three long and excellent reviews, one of which is upon the Life and Services of Dr. Drake, headed by a fine engraving of that gentleman. Among its original articles, is a valuable paper on Vesico-Vaginal Fistula, by Dr. Bozeman, of Montgomery, Alabama, with an account of seven successful cases, in all of which the new mode of suture invented by him, called the Button Suture, was used. The record department contains much interesting matter also.

Although it has given us much pleasure to speak thus highly of this new aspirant to professional favor, we consider it to be equally our duty as journalists, to record our disapprobation of many of the sentiments expressed in its editorials, in several of which there is manifested a spirit of hostility to Eastern journalists, for which we are entirely unable to account. "We do not aspire," says the first of these, "to the censorship of the medical literature of this country, or even the directorship of those who kindly place themselves within the range of our journal, but we are determined that so long as we live, and with whatever strength we possess, we shall cry out against the servile bending of the knee to the English Baal of medical criticism so common in some sections of the country, more especially in that part which was the first boldly to throw off the political yoke that England had placed upon our necks. We congratulate our Southern and Western brethren in their comparative freedom from this shameful idolatry, and we hope we have good reason to believe that they never will submit to its degrading influence."

We sincerely regret to see such sweeping imputations, and vague as they are sweeping, charged against any portion of the union. They are provocative of a spirit, that, largely indulged in, cannot but exercise a most injurious influence upon the good feeling which at present exists among all the journals of our country. Such appeals to sectional prejudice we know, are not uncommon in political harangues; we consider them out of place, however, in a respectable and scientific journal, one of whose endeavors, and not its least one, should be the maintenance of a friendly and fraternal feeling between the members of a common profession. The charge, which we fully believe to be unfounded, is the more remarkable, coming as it does from one who has so little to complain of in the manner in which he has been treated by the very critics whose good nature and sense of justice he thus takes the earliest opportunity to wound.

In another editorial, the Boston Medical and Surgical Journal, and by implication all other journals who have copied the article, are charged, when noticing the recent researches of M. Brown-Séquard, with ignoring Dr. Bennett Dowler's claims to discoveries made upward of twelve years since, upon the functions of the spinal nerves. We have looked through several recent American works on Anatomy and Physiology in relation to this subject, and in none of them have we found any mention made of Dr. Dowler's views. All of them, in fact, agree with Dr. Richardson, the junior editor of the Louisville Review, who makes the following statement in his Elements of Human Anatomy, (1854:) "Each spinal nerve originates from the side of the cord by two distinct parts or roots, called from their relative position the anterior and posterior roots; of these, the former is entirely motor and the latter sensory in its function." It would be preposterous to suppose that there existed a combination among all the writers of these works to reject Dr. Dowler's views; for reasons best known to themselves, they took no notice of them. Under these circumstances, we do not see why the charge mentioned above should be specially aimed against an Eastern Journalist.

We hope that the freedom with which we have expressed ourselves on this subject will not be misconstrued. No one has a higher respect than ourselves for the gentlemen who occupy the position of editors of the Louisville Journal. The profession is largely indebted to the senior editor in many respects; as author of various treatises his name is favorably known throughout the world; we hear with pleasure also, that his lot will hereafter be cast among us; may we trust that a better acquaintance with us will have the effect of dispelling the mists that distort his editorial vision upon the subjects we have above referred to.

New York Hospital.—Dr. J. G. Cheeseman has resigned his situation as surgeon of this institution, having served it faithfully, for the period of thirty six years.

—

Dr. Charles E. Ware has been appointed, by the trustees of the Massachusetts General Hospital, to fill the vacancy in the board of physicians occasioned by the resignation of Dr. M. S. Perry.

—

Latest Parisian Notions on the Administration of Chloroform—*From a Correspondent.*—At a lecture delivered by M. Grisolle, on the 8th inst., at the l'Ecole de Medicine, Paris, on the subject of anæsthetic agents, more particularly chloroform, and the accidents liable to occur from their employment—he stated, that the most troublesome result accompanying the use of chloroform, was spasmodic coughing; the most dangerous result was syncope. The principal characteristics of the latter effect, when produced by this agent, were the extreme suddenness of its occurrence, and the difficulty, or rather impossibility, owing to the patient being at the time in a state of anæsthesia, of applying any of the stimulants ordinarily used in syncope, as under the circumstances they have no effect. After citing all the methods that have been tried for the recovery of patients apparently dead from the effects of chloroform, he concluded, that decidedly the best was the attempt at restoring respiration by the operator blowing into the patient's mouth, his own being directly applied to it; and, as the tongue is liable to fall back upon the glottis, and thus impede the entrance of air into the larynx, that no hesitation should be had in transfixing it with a ligature in order to pull it forward. [Or still better, as Mr. Syme long ago recommended, seize hold of the tongue with the bull-dog forceps.] M. Grisolle considered that in those cases where the circulation was weak, ether was perhaps to be recommended in preference to chloroform, as having a more stimulant effect, and, consequently, less chance of inducing syncope. In closing his remarks, however, he stated that all anæsthetics must be regarded as unsafe agents, and not to be prescribed except in severe cases, and by no means to be indiscriminately employed in trifling cases, such as extraction of teeth, etc.—*Edinburgh Medical Journal.*

—

Revolution among the Eclectics.—The "Eclectic Medical Institute" in Cincinnati has been the scene of a riotous demonstration, in consequence of a quarrel among the members of the faculty It seems that a Dr. R. S. Newton had rendered himself obnoxious to the other professors, in consequence of some reflections upon them in the "*Eclec-*

*tic Medical Journal*," conducted by him. He was joined by Dr. O. E. Newton and Dr. L. Freeman, the alliance of the latter arising in consequence of "a difficulty in relation to a female student." Against them were arrayed the rest of the faculty, Drs. Cleaveland, Sherwood, Buchanan, Hoyt and King. The Newton faction forcibly entered the Institute in the night, and endeavored to take possession, but were resisted by Dr. Cleaveland and the others. Dr. C. was attacked "by the Newtons, Dr. Freeman, Dr. F.'s brother, a medical student, Dr. Newton's Irish boy Dan, and other parties, who threatened to kill him." A general fight ensued, with clubs and other weapons, but no one was injured. Dr. Newton and his party retreated up stairs, and occupying the lecture rooms, prepared for a defence. Dr. Sherwood, and a number of students and the police, took possession of the lower story. Dr. Newton's party wore afterwards discovered "attempting to get a small cannon in the back way, and the police captured it." The Newtonians had bedding, food, wine and lager beer, and were determined to stand a regular siege. At the last accounts, things were in *statu quo*. In the language of the Cincinnati *Daily Freeman*, to which we are indebted for the above facts, "we wait with anxiety for the result."

*Boston Med. and Surg. Journ.*

—

Death of Dr. Warren.—Dr. John Collins Warren, the eminent Boston surgeon, died on the 4th of May, in the 79th year of his age. His death could not be attributed to any distinct disease; his health had been bad for several years, and his vital powers were finally exhausted, without any important local affection. Dr. Warren was born in Boston August 1st, 1778. His father, Dr. John Warren, was a distinguished surgeon, and his uncle was the General Warren, the martyr of the revolution, whose name is so closely associated with the battle of Bunker Hill. Dr. Warren graduated at Harvard College in 1797. He pursued his professional studies in London, being a pupil of William Cooper, and subsequently of Astley Cooper. In 1806, Dr. Warren was appointed Adjunct Professor of Anatomy and Surgery at Harvard College, and in 1816, he succeeded his father in the full professorship. He held the chair of Anatomy until the year 1847. He succeeded Dr. James Jackson as president of the Massachusetts Medical Society in 1832, and filled this office four years. In 1803, he married Susan Lowell Mason, by whom he had six children. She died in 1841. In 1843, he married Anna Winthrop, by whom he had no issue. She died in 1850. Dr. Warren was a friend of the temperance movement, and for a long time presided over the Massachusetts temperance society. At the organization of the Massachusetts general hospital in 1817, Dr. Warren was elected surgeon to this institution—a post he retained until a few years before his death. At the time of his death he was emeritus professor of Anatomy and Surgery in Harvard College, president of the Boston Society of Natural History, a member of the American Philosophical Society, of the Philadelphia Academy of Natural Sciences, of the Academy of Naples, and the Medical Society of Florence, an honorary member of the Medico-Chirurgical Society of London, and a corresponding member of the Royal Academy of Medicine of Paris.—*Virginia Medical Journal.*

---

Death and a Will.—Vidal (de Cassis) died at Paris, in April last, In our own country, Dr. J. G. Percival, the poet, and Dr. John C. Warren, of Boston, are also gone. This last gentleman left a singular will, requiring his body to be placed in a tomb, with the usual ceremonies, and that the next day *after* it should be taken from the tomb, and an examination made, to ascertain the cause of certain obscure symptoms of disease under which he had for a long time labored. Then the will directs that his bones shall be macerated, wired, and hung up in the Museum of the Medical College in Boston. The will was peremptory. We learn that everything has been done as he directed, and that his bones are now macerating. There was found to be malignant disease of the stomach.

We confess that we feel a shudder creep over us when we think of this will. Had science required it we should have admired the self-sacrificing spirit which prompted it, but no such demand exists which could not be carried out infinitely better without shocking the feelings of any one. It is to be hoped that the trustees will put the skeleton into a case entirely of wood, at least till all of his friends have gone, and are no longer shocked by the sight. One thousand dollars were left by him for publishing his biography. One thousand dollars more to keep his tomb in repair forever. But *why* one does not see. *Nothing* was left to any hospital or other charitable institution.—*American Medical Monthly.*

---

Several specimens of Mathey-Caylus's preparations, consisting of capsules of Copaiba and Cubebs, Copaiba and Citrate of Iron, Copaiba and Rhatany, &c., have been left with us for trial. The peculiar advantages of these capsules are that "Although smaller by one half than the gelatine capsules, they contain as much Copaiba, owing to their envelopes being much thinner. In fact, the Gluten Capsule weighs hardly two grains and a half, whilst the gelatine capsule weighs at least twelve grains—a fact which may be easily verified."

We have had no opportunity for using any of these preparations ourselves; several of our friends, however, who have, speak very warmly in their favor.

